# Atlas-Free Surface Reconstruction of the Cortical Grey-White Interface in Infants

**DOI:** 10.1371/journal.pone.0027128

**Published:** 2011-11-16

**Authors:** François Leroy, Jean-François Mangin, François Rousseau, Hervé Glasel, L. Hertz-Pannier, Jessica Dubois, Ghislaine Dehaene-Lambertz

**Affiliations:** 1 INSERM, Cognitive Neuroimaging Unit, Gif sur Yvette, France; 2 CEA, DSV, I2BM, Neurospin Center, Gif sur Yvette, France; 3 University Paris 11, Orsay, France; 4 IFR49, Paris, France; 5 LSIIT, UMR 7005, CNRS-Université de Strasbourg, Illkirch, France; University of Barcelona, Spain

## Abstract

**Background:**

The segmentation of the cortical interface between grey and white matter in magnetic resonance images (MRI) is highly challenging during the first post-natal year. First, the heterogeneous brain maturation creates important intensity fluctuations across regions. Second, the cortical ribbon is highly folded creating complex shapes. Finally, the low tissue contrast and partial volume effects hamper cortex edge detection in parts of the brain.

**Methods and Findings:**

We present an atlas-free method for segmenting the grey-white matter interface of infant brains in T2-weighted (T2w) images. We used a broad characterization of tissue using features based not only on local contrast but also on geometric properties. Furthermore, inaccuracies in localization were reduced by the convergence of two evolving surfaces located on each side of the inner cortical surface. Our method has been applied to eleven brains of one- to four-month-old infants. Both quantitative validations against manual segmentations and sulcal landmarks demonstrated good performance for infants younger than two months old. Inaccuracies in surface reconstruction increased with age in specific brain regions where the tissue contrast decreased with maturation, such as in the central region.

**Conclusions:**

We presented a new segmentation method which achieved good to very good performance at the grey-white matter interface depending on the infant age. This method should reduce manual intervention and could be applied to pathological brains since it does not require any brain atlas.

## Introduction

**Figure 1:**
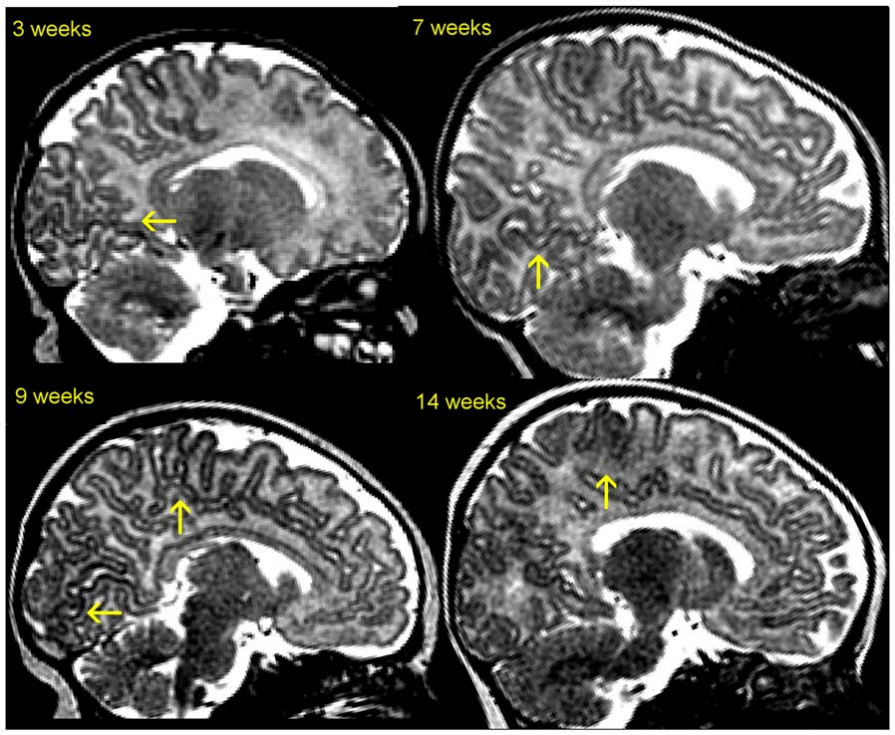
Infant brain maturation across age in T2w MRI. These sagittal slices show temporo-spatial variations of the on-going maturation processes near the inter-hemispheric plane. Areas of advanced maturation can be seen in primary cortices, such as along the calcarine sulcus and the central sulcus (*yellow arrows*).

**Figure 2:**
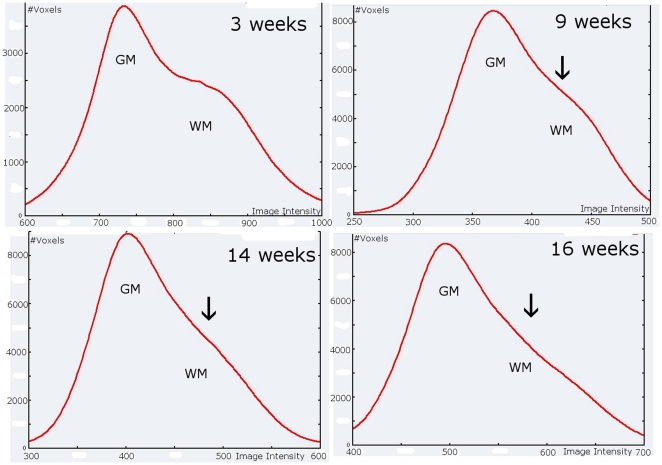
Frequency distributions (histograms) of brain tissue signal intensity according to age (3, 9, 14 and 16 week-old). Separate histogram modes of brain tissue disappear (*black* arrows) as the grey-white matter contrast decreases due to on-going maturation. GM: grey matter; WM: white matter.

Accurate reconstruction of the cortical border from MRI is an important issue in visualization, cortical mapping and quantitative brain analysis. It is highly challenging in infants because imaging an immature brain encounters several difficulties (figure 1). First, partial volume effect due to the small brain size associated with an already complex pattern of gyrification hampers cortex edges detection. Second, because of unmyelinated white matter, the contrast between grey and white matter is much weaker than the one typically found in adult MRI. Third, the human brain undergoes important and fast changes during the first post-natal year (e.g. the cranial perimeter increases by 0.5 cm per week). The grey-white matter contrast is so poor in T1w MR images that T2w MR images are preferred during the first months of life. However, as brain matures, tissue contrast decreases with age in T2w MR images (figure 2) whereas T1w MR images remain of poor quality [1]. Finally, maturation is not homogeneous across the brain, some areas showing intense myelination and proliferation of membranes (e.g. visual and motor areas) while others have a more protracted development (e.g. frontal areas) [2]. This inhomogeneity in maturation produces important variation in tissue intensity in MRI. Taken all together, these characteristics make segmentation of brain compartments a very difficult issue during the first year of life.

### Related work on brain segmentation

One approach to overcome segmentation issues in the developing brain is to use strong *a priori* information. Apart from a recent approach based on high-level anatomical knowledge from the subject itself [3], current neonatal brain segmentation methods have used either atlases or training datasets. Atlases were indeed very efficient to segment deep brain tissues [4], myelinated from unmyelinated white matter [5] or the relatively unfolded foetal brain [6]. As for the cortical folding patterns which are highly variable across infant brains, methods often built up highly specific atlases: Weisenfeld et al. [5] set up a large collection of scattered prototypes and selected those close to the subject anatomy; Shi et al. [7] used the highly contrasted T1w image of a one or two years-old child as a template for segmenting the T2w neonatal image of the same subject. When longitudinal data were not available, the same authors [8] devised another method which non linearly registered the to-be-segmented cortex to spatially close cortical segmentations from another neonate dataset. Finally, atlas-based priors were combined with local intensity information and cortical thickness constraint in a single level-set framework [9].

Besides, segmentation methods have to deal with spatial variations of tissue intensity caused by inhomogeneous maturation in neonatal brains, particularly the on-going white matter myelination. Several authors [4], [7], [10] referred to the Expectation-Maximization (EM) algorithm devised by van Leemput et al. [11], which iteratively corrects tissue for intensity inhomogeneity in MRI. Additionally, the EM scheme removes residual local noise based on Markov Random Field theory. Some authors have also modeled the irregular non-Gaussian white matter intensity using a set of non-parametric density functions [12] or split the brain into regions [4], [10]. More specifically to T2w MR images of the developing brain, the intensity of cerebro-spinal fluid close to cortex is similar to white matter intensity because of partial volume effect. Thus, cerebro-spinal fluid may be misclassified as white matter when detecting tissue near the cortical folding patterns. A knowledge-based strategy was introduced to reduce this effect and improve the classification [4], [5].

Finally, MRI resolution is a critical issue in infant brain segmentation. The developing brain is highly folded and the detection of its thin and twisted convolution patterns requires high T2w resolution. However, risk of motion in non-sedated infants prevents from long acquisition time. A trade-off between spatial accuracy and motionless acquisition might be a resolution near 1 mm in each spatial dimension, as it has been reported for segmenting the cortical ribbon [4], [6].

### Contribution of this paper

Our purpose was the cortical surface reconstruction across a large infant age range (chronological age from 1- to 4-month old) without any atlas requirement. It is highly challenging in older infants because of the persistent decrease of the grey-white matter contrast during the first year of life due to on-going tissue maturation.

We devised an atlas-free method because infant atlases are not yet of easy access and atlas-based strategies are often not well-adapted to the developing brain for several reasons. First, they require a careful selection of the infant template, because of the variability in brain shapes (particularly with the recent spread of plagiocephaly [13], i.e., an asymmetrical flattening of one side of the skull due to new sleeping habits required to prevent sudden infant death syndrome) and in the cortical folding patterns. Second, age-specific templates are required to deal with the fast and differential maturation and growing of the brain. It may be particularly tricky when the “chronological” age of the infant is different from its “maturational” age (chronological age corrected by gestational age at birth, for instance for full-term infants born at 37 weeks instead of 41 weeks). Finally, atlas-based approaches may not deal properly with pathological brains [14], such as malformations of cortical development.

Instead of the expectation-maximisation approach, we here initially corrected for spatial intensity inhomogeneities and then detected the cortical surface based on local priors which were not much sensitive to intensity inhomogeneities. We dealt with the issue of similar intensity in cerebro-spinal fluid and white matter close to cortex by warping two surfaces from each cortical side. These two surfaces competing with each other yield higher robustness than a single deformable surface because they efficiently build upon complementary information located on both sides of the grey-white matter interface. Both surfaces ultimately converged towards this interface whose curvature is smoother than the outer cortical surface and which is therefore easier to segment.

Specifically, our method relied on strong priors from the subject brain itself. Our first prior was the relatively steady thickness and darker intensity of the cortical ribbon in T2w images, compared with surrounding white matter and cerebro-spinal fluid intensities. It was detected using morphological top hats [15]. A second prior was the ridge segments of white matter intensity which were present in large brain regions as much as in narrow gyri for every level of maturation. We computed the mean curvature of isointensity surfaces to detect white matter ridges [16]. Detections of those priors were combined within a feature field. Finally, we applied a surface deformation approach onto the feature field in order to reconstruct the inner cortical surface. Mangin et al. [17] introduced a deformation method which preserved topology and removed local tissue noise based on Markov Random Field theory; such method produced faithful detections of the folding patterns in T1w MRI of adult brains. We applied this deformation approach to two converging surfaces, initialized on each side of the inner cortical interface and whose speeds were tuned according to both feature intensity and neighbourhood configuration.

To evaluate this method, we considered eleven one-to-four month-old-infants for which T2w sequences were acquired. Besides visual inspection of the results, we used two quantitative validations, first automatic vs. manual segmentations in 4 infant hemispheres, and then automatic segmentation vs. manually drawn sulcal landmarks in all infants. For the first validation, segmentation performance was based on standard evaluation methods [4], [10] and visual inspection. It gave a general idea of the method accuracy. To investigate this point further and also to enlarge the evaluation scope, we took advantage of a previous study based on sulcal characteristics in the linguistic network [18]. We compared automatic segmentations to manually drawn sulci in every infant. Because these primary sulci are robust landmarks of the infant brain, deviation relative to these landmarks informed us on the robustness of our method.

Finally, separate evaluation was done for younger infants aged less than two-month-old and for older infants aged more than two-month-old. Because maturation trajectories are different across cerebral areas, we expected more errors in older infants in primary cortices that myelinate early and fast during the first post-natal weeks. For example, the tissue contrast progressively disappears with age in the central region, which becomes dark both in grey and white matter. Thus, it was possible to relate segmentation performance to maturation state because these sulci have specific maturation profiles [19].

## Materials and Methods

### Infant MR Data Set

The dataset consisted of 11 healthy full-term infants from 3 to 16 weeks of chronological age (one to 4-month-olds). Infants were included in this study after their parents gave written informed consent. MR scans were acquired with T2 weighted fast spin-echo sequence (TE/TR = 120/5500 ms, echo train length = 17) on a 1.5T MRI system (Signa LX, GEMS, USA), using a birdcage head coil. Scans along the axial, sagittal and coronal directions were acquired for each infant. Spatial resolution was 1.04×1.04×2 mm (number of averages = 1, matrix size = 192×192, squared field of view = 20 cm). This set of data acquisition was approved by the following ethical committee: « Comité de consultation pour la protection des personnes se prêtant à la recherche biomédicale » (CCPPRB) of Kremlin Bicêtre (France); Protocol: #05-14; Promoter: INSERM; Main investigator: G. Dehaene-Lambertz.

### Preprocessing: Brain Reconstruction And Removal Of Skull, Cerebro-Spinal Fluid and Cerebellum

The image reconstruction algorithm, introduced in [20], computed a single image with high isotropic resolution (approximately 1×1×1 mm) for each subject from MRI acquisition sets. This method was automatic and its main steps were: multi-resolution slice alignment, contrast correction between native images, and super resolution reconstruction.

The Brainvisa toolkit [21] was used to strip the skull. We tuned the skull stripping tool, which had been originally designed for T1w images of the adult brain, to T2w intensity characteristics in the immature brain. This toolkit was also used to localize the inter-hemispheric plane. Then, most of the cerebro-spinal fluid was removed by hysteresis thresholding. The low and high thresholds were set manually for each infant. Indeed, most ventricular cerebro-spinal fluid needed to be carefully detected and filtered out because the boundaries with white matter were hardly noticeable in the feature field. Finally, cerebellum was manually segmented because its boundary is blurred near the cortex due to partial volume effect. At the end of this step, we obtained a brain mask for each hemisphere.

### Building the Feature Field using Local Contrast and Curvature Minima

We detected tissue features based on local contrast and geometrical tissue properties. Our detectors, namely morphological top hats and curvature minima, are local differential measurements and are therefore not much sensitive to intensity fluctuations across the brain. Besides, they would behave better than any contrast-based detector in weakly contrasted regions of the brain because they rely upon geometrical properties, namely cortical thickness and line ridges in gyri. These features were combined within a feature field which was intended to enhance contrast between white matter and cortex and therefore to increase the detection of the inner cortical surface.

**Figure 3:**
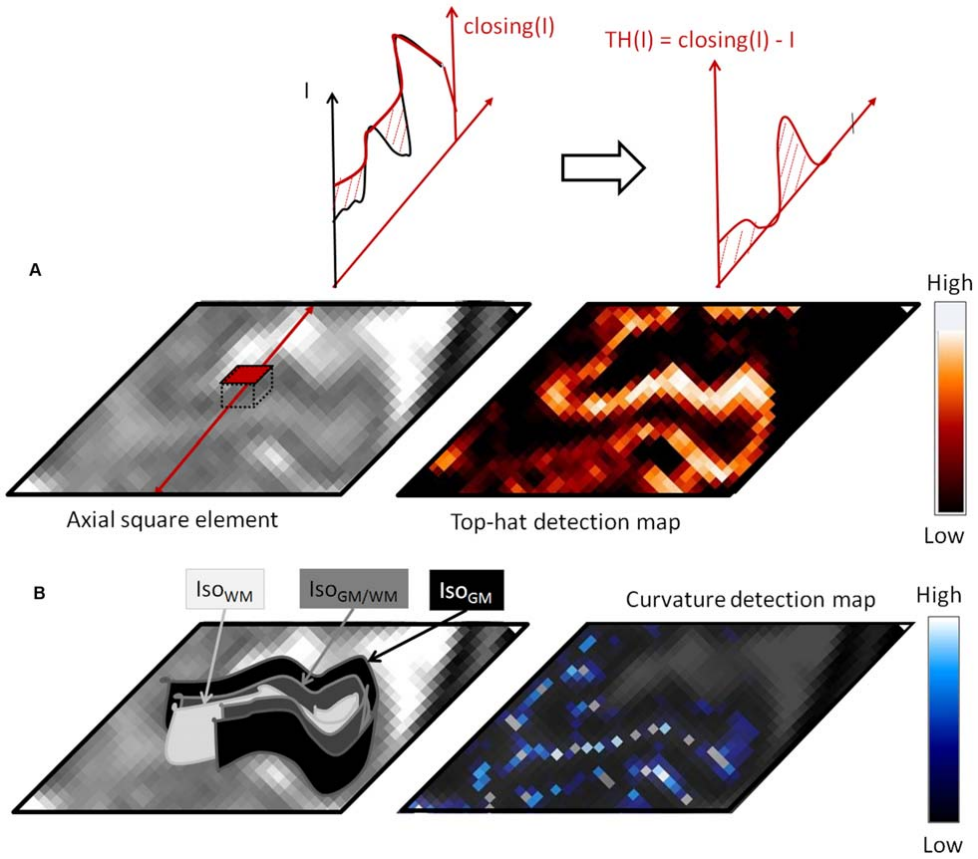
Description of morphological operators over a MRI axial slice including a cortical gyrus. A. Case of top hat detection of grey matter. A morphological closing is first applied to the image intensity using square elements such as the axial one shown. Results of the closing operation, namely *Closing(I)*, as well as the intensity profile (*I*), are depicted on the top left along a given sagittal cut. Top hat detection, i.e., *TH(I)*, which is the subtraction of *I* from *Closing(I)*, is shown on the top right. The detection map over the axial slice is shown with *red-white* color table. B. Case of curvature detection of white matter. Three portions of isointensity surfaces are shown on the left: a grey matter surface in *black*, an intermediate surface in *grey* and a white matter surface in *light grey*. When the isointensity surface is folding up such as the white matter one, curvature reaches minimal (negative) values. These curvature minima make up ridge lines, such as the gyral one shown on the curvature map with *blue-white* color table.

We first computed the mean curvature of isointensity surfaces in white matter, whose negative value was named MC^WM^ in this section. MC^WM^ is a differential characteristic of specific surfaces called isointensity surfaces. For a given intensity *I,* there is an isointensity surface in every MRI volume which sets apart voxels of lower intensity and voxels of greater intensity than *I.* We computed two differential characteristics of such surface, namely the two curvatures along its principal directions. Computation of principal curvatures was based on the implicit definition of the surface [16]. The mean of these principal curvatures has an intriguing property in brain MR images: its minima (negative) values, i.e., MC^WM^, correspond to ridges (gyri) of the folding patterns in T2w MR images (figure 3B) [16].

For a better understanding, let us pick up one MR slice of the image and a given gyrus within this slice. Now, let us imagine white matter intensity as the third dimension of the picture. The gyrus would be seen as a mountain with the darker cortical ribbon being the bordering valley. Isointensity lines in white matter would behave as the hill level sets. Mean curvature along these lines would be smooth everywhere except at the mountain crest where it is no longer defined (mathematical singularity). In that case, curvature minima, e.g. MC^WM^, would be the line ridge of the mountain.

Then, we applied morphological top hats [15] to detect the cortical ribbon, which has a relatively steady thickness and darker intensity than surrounding white matter and cerebro-spinal fluid intensities (figure 3A). These characteristics of the cortex are exactly those used to detect roads in remote-sensing applications, i.e., a curved and narrow dark material compared with surrounding natural landscapes. Morphological top hats have been highly efficient to detect roads in optical and radar images [22]. We applied this approach to the 3D case.

Specifically, top hats were based either on a morphological closing (grey matter) or on an opening (white matter). A closing (opening) top hat consists of detecting dark (bright) areas of the image intensity function whose sizes fit those of pre-defined simple shapes called structuring elements, respectively. Structuring elements were squares in our approach. We applied a set of nine analog top hats in nine planes whose orientations were the sagittal, axial, coronal axes and bisecting directions. In each plane, each top hat probed the image intensity function with a set of square elements. Square size was set according to thickness variations of each tissue: side-lengths of 2 or 4 mm for grey matter detection and side-lengths of 4, 7 or 14 mm voxels for white matter detection. Finally, we computed the maximum detection across the nine directions and for both square sizes.

For a given square size *s*, top hat equations were given as follows:
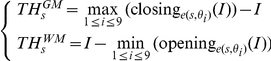

*I* stands for MRI intensity and e(*s*,θ_i_) are the 9 square structuring elements with side length *s* and whose orientations θ_i_ are the sagittal, axial and coronal axes and 6 bisecting directions.

These equations enabled to compute the weighted average of the grey matter (white matter) outputs across all square sizes, namely TH^GM^ (TH^WM^), respectively.

**Figure 4:**
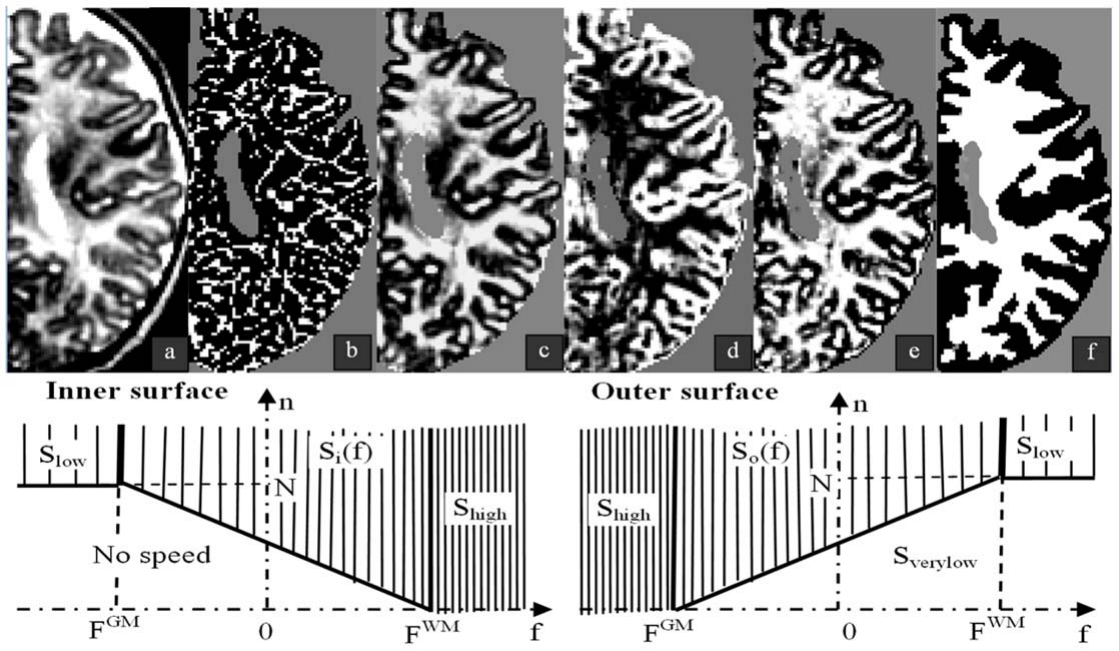
Segmentation steps and speeds of surface deformations. Upper row: a: MR axial slice of a 9-week-old infant; from *b* to *d*: brain mask in *grey* and detection outputs in *white*; *b:* MC^WM^; *c:* TH^WM^; *d:* TH^GM^; *e:* feature field with cortical grey matter in *black* and white matter in *white*; tissue contrast is enhanced over the brain; *f*: white matter segmentation. Lower row: Speed variations in second round deformation according to feature value *f* and the number of neighbors *n*. S_high_: high speed; S_low_: low speed; S_verylow_: very low speed (outer surface only); When contrast is weak, i.e., f in [F^GM^, F^WM^], *S_i_(f)* and *S_o_(f)* speeds are finely tuned according to both feature and neighbor configuration. Speed equations are given in table 1 and 2 for the inner and outer surfaces, respectively.

Finally, the outputs of TH^GM^, TH^WM^ and MC^WM^ were normalized (figure 4) and combined within the feature field. Normalization was linear for mean curvature outputs and sigmoidal for top hats. Because ridge detectors are more specific than top hats, sigmoidal normalization levels off accuracy performance across detectors. The feature field was produced by weighted average of all normalized detections, such as follows:
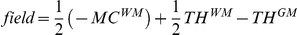
Note that white matter has positive values in the feature field while cortex has negative values. An example of feature field is depicted in figure 4.

### Homotopic Deformation of Coupled Surfaces

Two surfaces were initialized on each side of the inner cortical interface within the feature field. The surface model is a closed set of connected voxel facets. It defines the interface between two volumetric regions, namely the inner and outer volumes. The dilation of the inner volume inflates the inner surface whereas the dilation of the outer volume deflates the outer surface.

Surface evolution is based upon the energy minimization framework defined in [23]. The grey-white interface is defined as the minimum of a global energy U, which is the sum of a data driven component U_D_ and a regularization component U_R_. According to section 4.2.3 in [23], U_D_ is the sum of potentials attached to order one cliques, whereas U_R_ is constituted by Ising model attached to order two cliques. The minimization of U is performed using a deterministic algorithm similar to the iterated conditional modes: during surface propagation, points are added to the region being dilated sequentially, an addition occurring each time it produces an energy decrease (ΔU<0). Although this approach gives only a local minimum of U, it turned out to be sufficient to obtain very good segmentation results in the adult brain [23].

The outer surface was set by the brain bounding box. Initialization of the inner surface resulted from a threshold of the feature field. The threshold was computed iteratively so that the inner surface area reached a given size. The initial inner size was common to all subjects because well-fitted surfaces had similar surface areas during the optimization round.

A first round of deformation was applied on both surfaces across the feature field. It was intended to move surfaces closer to each other while yet avoiding conflicting areas. We set two feature thresholds which bounded those areas, namely *F^GM^* and *F^WM^*. The inner surface inflated when feature value *f>F^WM^*; conversely, the outer surface deflated at the same speed when *f<F^GM^*.

A second round of deformation achieved the final convergence of the two surfaces. Coupled speeds were applied on outer surface deflation and inner surface inflation. Propagation stopped when the surfaces met, ideally at the inner cortical border.

In this round, deformation speed was regulated for dealing with conflicting regions, i.e., areas with irregular feature contrast. When speed was low (S_low_) at a given target location for either the inner or outer surface, say because of adverse feature contrast, speed was high (S_high_) for the other surface to favor its propagation towards that location. Thus, there was an indirect speed coupling between the inner and outer surfaces.

Speed was monitored by setting a “*potential to move*” *P* at each surface location, similar to gravitational potential energy in physics. At each deformation step, potential *P* was decreased according to speed (*S*), i.e., ΔP = P_0_ * (S/S_high_). Initial potential *P_0_* was the same across all surface locations. When *P* reached zero, propagation was triggered. Only one deformation step was enough for high speed in order to cancel potential and trigger propagation. However, when speed was low, potential decrease *ΔP* amounts to a fraction of *P* so that a few iterations were required for propagation. *P* was reset to *P_0_* after propagation.

Within the energy minimization framework, propagation speed is positive if and only if there is an energy decrease. This rule was strictly fulfilled for the inner surface. However, a very low but positive speed (S_verylow_) was applied when ΔU≥0 for the outer surface, in order to let the surface propagate over residual cerebro-spinal fluid voxels; Thus, the deflation of the outer surface did not stop when it reached a local energy minimum but only when it overlapped the inflating inner interface.

Speed range was common to both surfaces and speed variations depended upon both the feature value *f* and the number *n* of surface neighbors to the given target location. Speed variations are summarized in figure 4 and tables 1 and 2.

**Table 1:** Speed of the inner surface.

	Number of neighbors (n)		
Feature value (f)	n<N_I_(f)	N_I_(f)<n<N	n>N
f<F^GM^	No speed (0)		Low speed (S_low_)
F^GM^<f<F^WM^	No speed (0)	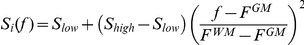	
f>F^WM^	High speed (S_high_)		

Speed (S_i_) as a function of the number of neighbors (regularization) and feature value (f) with 
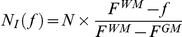
. See text for details.

**Table 2:** Speed of the outer surface.

	Number of neighbors (n)		
Feature value (f)	n<N_O_(f)	N_O_(f)<n<N	n>N
f<F^GM^	High speed (S_high_)		
F^GM^<f<F^WM^	Very low speed (S_verylow_)	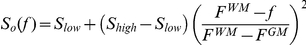	
f>F^WM^	Very low speed (S_verylow_)		Low speed (S_low_)

Speed (S_o_) as a function of the number of neighbors (regularization) and feature value (f) with 
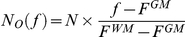
. See text for details.

Speed of the inner surface is high when it is likely white matter, i.e., f>F^WM^. When contrast is weak, i.e., f in [F^GM^, F^WM^], *S_i_(f)* speed is finely tuned according to both feature and neighbor configuration. Equation is given in table 1. When it is likely grey matter, i.e., f<F^GM^, surface deformation is either stationary (speed = 0) or small (speed = S_low_) only when there is enough surface neighbors (n>N).

As for the outer surface, speed is high when it is likely grey matter, i.e., f<F^GM^. When contrast is weak, i.e., f in [F^GM^, F^WM^], *S_o_(f)* speed is finely tuned according to both feature and neighbor configuration. Equation is given in table 2. When it is likely white matter (f>F^WM^), speed is set to either low (S_low_) or very low value (*S_verylow_*), to let the surface propagate over residual cerebro-spinal fluid voxels.

Finally, a topological constraint was added to the outer surface before propagation towards a given surface location (for both rounds of deformation). This requirement, which is called homotopy [23], preserved the spherical topology of the surface under deformation and prevented from tricky segmentation errors such as image handles. We applied our constraint to the outer surface which initially was the surface of a box and therefore had a spherical topology. Therefore, the spherical topology of the outer surface was held along the deformation process.

The topological constraint was assessed at each target location before a given step of deformation. Let us call *T* the target location, *S* the neighboring voxels belonging to the deformation surface and *B* the voxels which belonged to the background neighborhood of *T*. Sets *B* and *S* make a partition of *T* neighborhood. We then counted the number of connected sets of voxels in *S* and *B*. If *S* and *B* were both made of one and only one connected component, the topological requirement was fulfilled and the deformation towards *T* was triggered.

### Method Optimization and Robustness

The eleven brains in our infant dataset have been used as follows. We designed and trained our method based on two infant brains (4 and 11 week-old). The method was then optimized and tested on another set of three brains (7, 10 and 16 week-old). During optimization, speed parameters were set as follows: 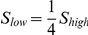; 
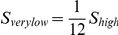
 and *N* = 22 for both surfaces, where N is a regularization threshold. F^WM^ and F^GM^ were set to 12% and 8% of highest and lowest feature values, respectively. The same parameters were used in all infant brains.

We believe that most segmentation parameters are weakly dependent on acquisition parameters. Top hat size *s* is mostly based on cortical thickness. The regularization factor *N*, related to neighborhood, accounted mostly for local tissue noise. If noise increases in some MR image, user would rather pre-process the data instead of tuning *N*, by applying some smoothing filter such as anisotropic diffusion filtering.

### Evaluation Measurements

To evaluate this method, we first compared automatic segmentations with manually segmented left hemispheres of four infants (3, 7, 9 and 14 week-old). Three out of these four infant brains have not been used during the training and testing stages (see previous section). We appraised segmentation results with standard evaluation tools, i.e., the Dice coefficient and the surface reconstruction error [4], [10]. It gave a general idea of the method accuracy.

Second, we compared automatic segmentations to manually drawn sulci in all eleven infants. Because these primary sulci are robust landmarks of the infant brain, deviation relative to these landmarks informed us on the robustness of our method.

Furthermore, results of these two evaluations were analyzed separately for younger infants (G1 group; 5 infants from 3 to 9 week-old) and older infants (G2 group; 6 infants from 10 to 16 week-old).

#### Manual segmentation of brain hemisphere

Four brain hemispheres were manually segmented. They were selected to span the whole age range, ie, 3, 7, 9 and 14 week-old. White matter segmentation was done in every axial slice and then checked in coronal and sagittal slices. We chose to assess our segmentation method across the whole hemisphere instead of a few slices per subject for the following reasons: first and foremost, grey-white matter contrast is changing across the brain because of tissue maturation (for instance, low contrast in medial occipital and central regions). Second, the complex geometry of the cortical folding patterns may impede deformation-based methods, such as in elongated and narrow gyri. Thus, evaluation across whole hemisphere gives useful information on how the algorithm deals with the full cortical geometry and the spatial variations of tissue signal. Finally, we used additional landmarks, i.e., manual sulcus drawings, to further evaluate our method in every brain.

#### Dice coefficient

The similarity between automatic (A) and manual (M) white matter segmentations was measured using the Dice similarity coefficient [24]; It measured the overlap between two regions and was given by Dice Coef. = 2*(A ∩ M)/(A+M).

#### Global accuracy at the grey-white interface

Distance gap between automatic and manual surfaces was a measure of the segmentation accuracy at the grey-white matter border and was therefore computed at each surface voxel. Because the grey-white interface includes both cortical and subcortical areas, we measured segmentation performance both over the whole interface and specifically over the cortical areas for which our algorithm was initially designed.

#### Measurements at sulcal landmarks

**Figure 5:**
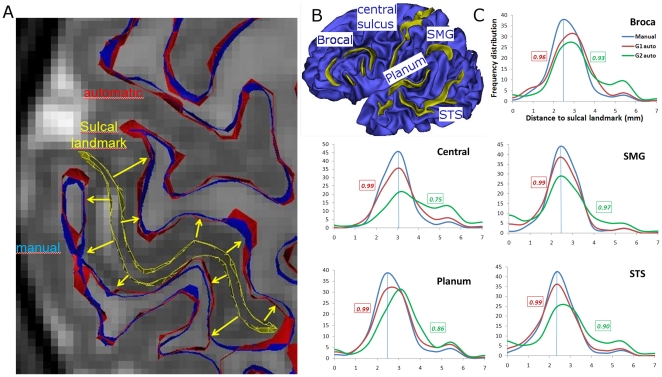
Validation against sulcal landmarks. A: Mesh cuts overlaying an axial slice; Sulcal landmark in *yellow*, and the automatic (in *red*) and manual (in *blue*) cortical segmentations. *Yellow* arrows are vectors normal to the sulcal surface, along which distances to segmentations are computed. B: Manually delineated sulci (in *yellow*) and the underlying inner cortical mesh (in *blue*) in a 7 weeks old infant. C: Frequency distributions of distances from sulcal landmarks to manual segmentations for the four selected infants aged 3, 7, 11 and 14 weeks old (in *blue*), to automatic segmentations of the younger infant group G1 (in *red*) and to automatic segmentations of the older infant group G2 (in *green*). Correlation coefficients between manual and automatic distributions are shown for both G1 and G2 groups. SMG: supra-marginal gyrus; Planum: *planum temporale*; STS: superior temporal sulcus.

We measured the distance between segmentations and manually segmented sulci in each infant brain (figure 5A). Distance from manual segmentation to sulcal landmark was used as a reference. Automatic segmentation was compared with manual segmentation based on their respective distance to these landmarks. Deviation from this reference informed us on the performance of our method. Under-segmentation at a given landmark, e.g. an incomplete gyrus, would increase the distance to landmark relative to reference. Conversely, over-segmentation, e.g., a missed sulcus, would decrease the distance to landmark relative to reference. We considered only left hemispheric segmentations because only left hemispheres were manually segmented.

H.G. manually drew the central sulcus, the superior temporal sulcus, the inferior frontal sulcus, Broca's rami, the *planum temporale*, as well as the sulci bordering the supra-marginal gyrus (figure 5B). This set of sulci is spread across the lateral parts of both hemispheres and includes structures with different maturation trajectories [19]. Drawings of sulci were validated by an expert neuroanatomist (L.H.P.). These sulci are robust landmarks which extend over a large amount of the lateral cortex and comprise cortical regions at different maturational stage. The central sulcus and Heschl's gyrus are primary cortices, thus being more mature than other regions [19]. They are also of different size. The central sulcus and the STS are large whereas Broca's rami are small and inconstant at these ages [18]. Thus this set represents quite well the characteristics of the infant brain.

Specifically, distance was computed between every landmark voxel and a given segmentation. The set of distances was then used to build a distribution of distance frequencies. Frequency distributions were averaged over all manual segmentations to provide with a reference distribution at a given landmark (*blue* curves in figure 5C). Frequency distribution for automatic segmentation was then compared to this reference distribution using the correlation coefficient.

Some of these histograms, including those related to manual segmentations, had heavy tails. It can be explained by the increasing distance between segmentation and landmark in the sulcal regions closer to the skull where the folds widen.

## Results

### Validation against Manual Segmentations of Brain Hemispheres

**Figure 6:**
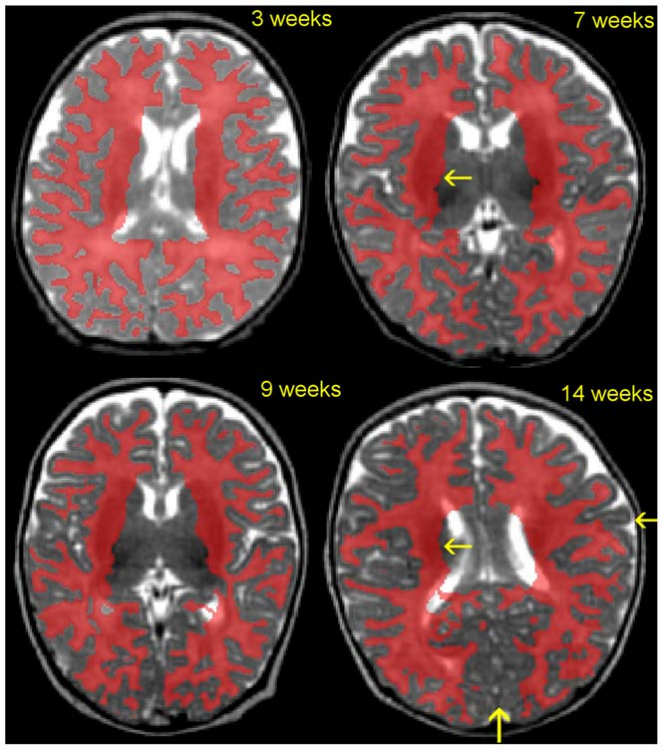
White matter segmentation across age. Segmentations are shown *in red* over axial MRI slices. Despite highly folded patterns, segmentation is accurate at the cortical border for younger infants (3, 7 and 9 weeks); however, segmentation at the subcortical boundary needs improvement (*yellow arrow*). As for infants older than two months old (14 weeks), segmentation performance decreases in a few areas because of on-going maturation, such as along the calcarine sulcus and the central sulcus (*yellow arrows*).

**Figure 7:**
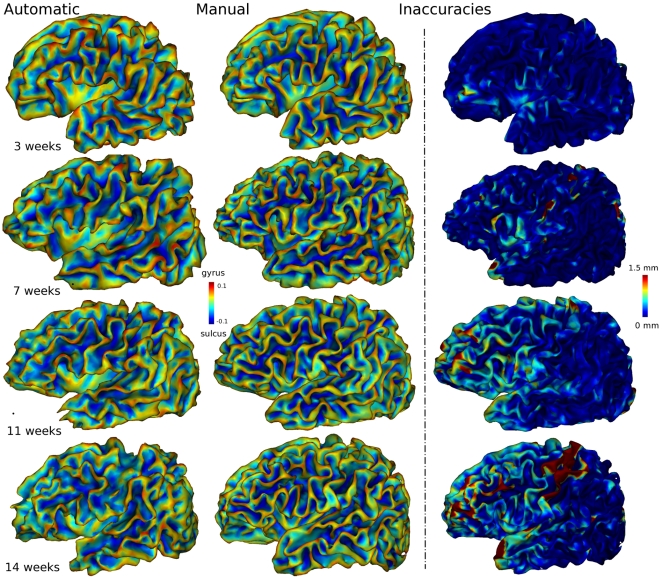
Validation of automatic segmentations against manual segmentations in 4 left hemispheres from infants with increasing ages (3 to 14 weeks). The two left columns show 3D renderings of the cortical surface reconstruction for automatic and manual segmentations. Color outlines surface curvature: gyri *in red* and sulci *in blue*. Method inaccuracy, i.e., distance from automatic to manual segmentation, is shown in the right column. Color map shows accuracy *in blue* and inaccuracy *in red* (in mm, up to 1.5 mm). Thus, *yellow-red* areas in the right column point to locations of segmentation inaccuracy. Inaccuracy patterns can further be analyzed in the two left columns by comparing the automatic surface to the manual one at those locations. See also in figure 1 for MRI slice samples of these four babies.

We first evaluated our method by comparing automatic segmentations with manual segmentations of four whole left hemispheres. White matter segmentations are shown in figure 6. Surface reconstructions are presented in figure 7, together with manually segmented hemispheres.

The Dice coefficient mean and standard deviation were equal to 0.87+/−0.05 over cortical areas (0.83+/−0.04 including subcortical tissue): in G1 (5 younger infants from 3 to 9 weeks old), Dice = 0.89 (0.85 including subcortical tissue); in G2 (6 older infants from 10 to 16 weeks old), Dice = 0.82 (0.79 including subcortical tissue).

The surface reconstruction error was 0.36+/−0.67 mm across the cortical border (0.51+/−1.1 mm including subcortical interface): in G1, error = 0.33 mm (0.49 mm including subcortical interface); in G2, error = 0.47 mm (0.53 mm including subcortical interface). In each group, standard deviation of errors was equal to the whole dataset deviation.

An overview of the method performance is shown in figure 7, which compares 3D renderings of automatic segmentations with manual ones and points to locations of inaccuracy. Visual inspection of the surface reconstruction identified only minor errors for the two youngest hemispheres (<2 months old in the two first rows of figure 7): a few inaccuracies (≈1.5 mm) at the temporal pole, along the calcarine fissure, as well as within the post central gyrus; less intense but more diffuse errors in the anterior part of the frontal lobe. As for the two older infants (≥2 months in the two last rows in figure 7), inaccuracies spread across prefrontal regions, the temporal pole, the medial occipital regions, and along the central sulcus.

### Validation against Sulcal Landmarks

There was a good performance for the younger group (G1, correlation = 0.98+/−0.01): frequency distributions of G1 segmentations are very close to those of manual segmentations (figure 5C). G1 distribution peaks match the manual ones, which are estimates of the half size of the sulcal width.

There was a relative decrease of performance for the older infant group (G2, correlation = 0.88+/−0.08). In G2, we observed a few heavy tailed distributions which can be explained by missing parts of gyral white matter (under-segmentation), most probably near gyral crowns (Central and Broca's distributions, figure 5C). Inaccuracies near the central sulcus and in Broca's region were also reported during visual inspection in the previous section.

## Discussion

In this article, we presented a segmentation method implemented for T2w images of the developing infant brain and we carried out an extensive validation. In addition to global evaluation based on standard tools, additional segmentation appraisal was provided in many brain regions with both inaccuracy maps for manually segmented hemispheres and measurements with robust landmarks in every subject.

Validation methods confirmed a good performance of the proposed approach in infants younger than 2 months old (G1). Only a few residual errors remained in anterior prefrontal regions, across the post-central gyrus, along the calcarine fissure, and at the temporal pole. Our Dice coefficient value (0.89) was higher than Prastawa et al.'s coefficient [10] but these authors computed a refined segmentation which additionally classified myelinated and unmyelinated white matter; It was equal to Shi et al.'s one [8] who used a dedicated phased array coil. Besides, our cortical interface accuracy was higher than in Xue et al. (reconstruction error: 0.73 mm in [4]); however, these authors extracted both the inner and the outer cortical surfaces and reported higher accuracy at the outer border than at the inner cortical surface.

Segmentation performance decreased in older infants (G2). Evaluation differences in younger and older infants are concordant with our own observations (figure 1 and 2). The decrease in the Dice coefficient and surface reconstruction accuracy (hemisphere-based evaluation) together with the decrease in the correlation coefficient (landmark-based evaluation) strongly suggest that there is a significant loss of tissue contrast between the second and the fourth month of life.

To our knowledge, our method is one of the only two published methods dealing with infants older than two months. Furthermore, the other one, by Shi et al [25], was related to longitudinal data which are yet unusual in infant imaging.

Visual inspection revealed that inaccuracies spread across prefrontal regions, the temporal pole, the medial occipital regions, and along the central sulcus. Validation using sulcal landmarks confirmed under-segmentation in the pre-central and post-central gyri, the supra-marginal gyrus and the inferior prefrontal regions, most probably at the gyral crowns. Two opposite effects may explain these segmentation errors. First, primary cortices mature before associative cortices, i.e., the sensory-motor regions along the central sulcus and the primary visual cortex along the calcarine fissure are more mature than most other parts of the brain [2], [26]. Thus, segmentation was hampered in these regions because the tissue contrast was decreasing and tissue intensity weakened. Second, there is a posterior-anterior maturation axis and both prefrontal regions and the temporal pole are among the most immature regions [27]. Thus, the thin cortical ribbon and the underlying unmyelinated white matter produced narrow gyri in T2w MRI in which partial volume effect is strong and tissue contrast is poor.

Future directions would include the use of multispectral data for increasing tissue contrast, such as T1w and T2w MRI [25], as well as an interactive user interface to deal with segmentation inaccuracies. We believe that user expertise could greatly improve the segmentation performance with a fair amount of time because segmentation inaccuracy was restricted to a limited set of brain regions [14].

To conclude, we presented a new method for segmenting the grey-white matter interface of infant brains. It was based upon a broader characterization of tissue properties in T2w MRI of the developing brain. Local contrast features were combined with geometrical tissue properties, i.e., line ridge segments in white matter and steady cortical thickness. Tissue contrast, which is lower in the most mature brain regions in T2w images, was enhanced in our designed feature field. Moreover, two converging surfaces, located on each side of the inner cortical border, reduced localization errors in areas with weak feature contrast. This method was automatic to the extent that, apart from its preprocessing step, a common set of parameters was used over the whole data set. Besides, no brain atlas was required, which could be particularly useful for pathological brains where lesions or malformation (e.g. dysplasia, corpus callosum agenesia) occurred. These brains might be too far from normal atlases to be successfully segmented using top-down information. Finally, this method achieved high performance at the grey-white matter interface for babies younger than two months old and would require manual correction only in a limited set of brain regions (the most mature) for older infants.
